# Lysine 473 Regulates the Progression of SLC7A11, the Cystine/Glutamate Exchanger, through the Secretory Pathway

**DOI:** 10.3390/ijms251910271

**Published:** 2024-09-24

**Authors:** Anna Koppin, Leah Chase

**Affiliations:** 1 Departments of Biology and Chemistry, Hope College, Holland, MI 49423, USA; koppi033@umn.edu; 2 Neuroscience Program, Departments of Biology and Chemistry, Hope College, Holland, MI 49423, USA

**Keywords:** SLC7A11, xCT, System x_c_^−^, endoplasmic reticulum acetylation, trafficking, glycosylation

## Abstract

System x_c_^−^, the cystine/glutamate exchanger, is a membrane transporter that plays a critical role in the antioxidant response of cells. Recent work has shown that System x_c_^−^ localizes to the plasma membrane during oxidative stress, allowing for increased activity to support the production of glutathione. In this study, we used site-directed mutagenesis to examine the role of C-terminal lysine residues (K422, K472, and K473) of xCT (SLC7A11) in regulating System x_c_^−^. We observed that K473R exhibits loss of transporter activity and membrane localization and is 7.5 kD lower in molecular weight, suggesting that K473 regulates System x_c_^−^ trafficking and is modified under basal conditions. After ruling out ubiquitination and neddylation, we demonstrated that unlike WT xCT, K473R lacks N- and O-glycosylation and is sequestered in the endoplasmic reticulum. Next, we demonstrated that K473Q, a constitutively acetylated lysine mimic, also exhibits loss of transporter activity, decreased membrane expression, and a 4 kD decrease in molecular weight; however, it is N- and O-glycosylated and localized to the endoplasmic reticulum and Golgi. These results suggest that acetylation and deacetylation of K473 in the endoplasmic reticulum and Golgi, respectively, serve to regulate the progression of the transporter through the biosynthetic pathway.

## 1. Introduction

System x_c_^−^ is a membrane transporter that plays a central role in the cellular antioxidant response [1,2], including protection from ferroptosis, an iron-dependent, non-apoptotic mechanism of cell death associated with numerous disease pathways [3]. This membrane transporter exchanges intracellular glutamate for extracellular cystine, and the internalized cystine is rapidly reduced to cysteine, which is required for the synthesis of the endogenous antioxidant glutathione. Given that cysteine is the rate-limiting reagent for glutathione synthesis, the proper regulation of System x_c_^−^ is essential for the maintenance of glutathione levels and prevention of oxidative stress and ferroptosis [3].

Recently, System x_c_^−^ has been shown to be acutely regulated by oxidant exposure [1]. Within 10 min of exposure to physiologically relevant levels of hydrogen peroxide, System x_c_^−^ traffics to the plasma membrane, resulting in a doubling of transporter activity, recovery of cellular glutathione levels, and re-establishment of oxidative balance within the cell [1]. Specifically, hydrogen peroxide appears to increase the transporter delivery rate and decrease the internalization rate from the membrane, resulting in an overall increase in cell surface localization. However, the mechanism by which hydrogen peroxide influences the trafficking of the transporter is not yet understood.

Examples of dynamic regulation of the trafficking of membrane proteins to and from the cell surface are as numerous as the signals that appear to regulate them [4,5,6,7,8,9,10]. For example, G-protein-coupled receptors exhibit downregulation after prolonged exposure to agonists, which triggers an increase in receptor internalization [11], and the epithelial sodium channel is activated by aldosterone [12], leading to a reduction in the rate of internalization of the channel from the plasma membrane. In nearly all cases, these changes in trafficking result from signal-induced changes in post-translational modification (PTM) of trafficking motifs within the N- or C-terminus of the membrane protein. Ultimately, the shift in PTM status of the protein alters its interaction with trafficking proteins within the cell, directly impacting protein delivery and/or internalization rates.

While there have been numerous studies that have detailed the transcription factors that regulate the expression of System x_c_^−^ [13,14,15,16,17,18], surprisingly have examined the acute, reversible regulation and trafficking of this transporter. System x_c_^−^ belongs to the family of heterodimeric amino acid transport (HAAT) proteins (SLC7 gene family) that includes nine different members with unique amino acid substrate specificities (for review, see [19,20,21]). Like most members of the HAAT family, System x_c_^−^ is composed of two proteins, a 50 kD light chain protein xCT (SLC7A11) that is linked through a disulfide bond to a 98 kD type II glycosylated heavy chain protein 4F2HC (SLC3A2) [22,23]. Each member of the HAAT family has a unique light chain that confers substrate specificity, while the role of the heavy chain is not well understood [24,25,26,27].

Studies focused on identifying PTMs of xCT and 4F2HC are lacking, and to date, no definitive trafficking motifs within xCT have been identified. Serine 26 of xCT is phosphorylated by the mammalian target of rapamycin 2 (mTORC2), leading to the inhibition of transporter activity, perhaps through changes in membrane localization [28]. Very recently, xCT was reported to be palmitoylated at cysteine 327, which appears to increase the stability of the protein, perhaps by reducing its ubiquitination [29]. Lastly, five phosphorylation sites were identified in the extracellular domain of 4F2HC, but these motifs appear to regulate cell–cell interactions [30]. Thus, our understanding of the role PTMs play in the regulation of System x_c_^−^ is poor relative to other families of membrane proteins.

**Figure 1:**
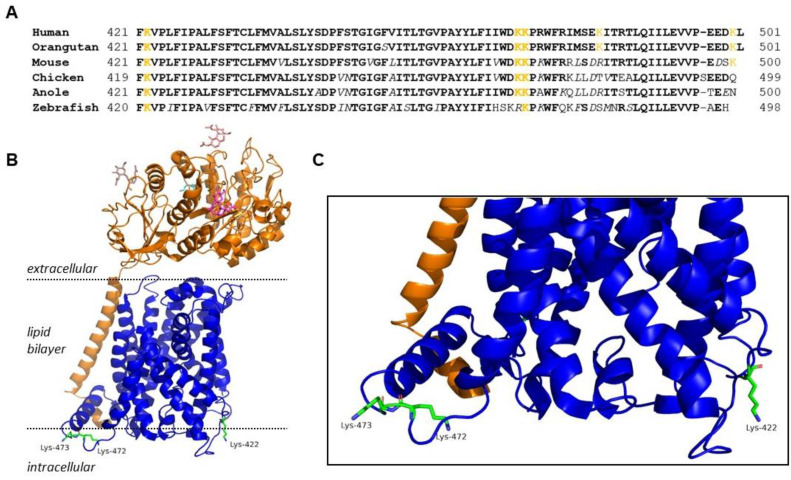
(**A**) A cross-species amino acid sequence comparison of the C-terminus of xCT showing three highly conserved lysine residues (K422, K472, and K473). Lysine residues are highlighted in orange; bold type indicates fully conserved amino acids (identical across species); white italics indicate conservative amino acid substitutions. (**B**) Cryo-EM structure of System x_c_^−^. xCT is shown in blue, and 4F2HC is shown in orange with K422, K472, and K473 side chains of xCT detailed on the cytoplasmic surface (PDB 7P9V) [36]. (**C**) Magnified view of cytoplasmic interface of xCT and conserved lysines.

Therefore, the primary objective of this study was to use site-directed mutagenesis to identify trafficking motifs within xCT so that we can better understand its regulation. Since many trafficking motifs are typically found within the internal N- or C-terminus of proteins, we chose to focus on the C-terminus of xCT, given that it is more highly conserved across species relative to the N-terminus (Figure 1). We reasoned that the high degree of conservation increased our likelihood of identifying trafficking motifs. In addition, we chose to focus on lysine residues exclusively in this study. Lysine residues are common sites of PTM that are known to impact protein trafficking, including ubiquitination, neddylation, SUMOylation, and acetylation [31,32,33,34,35]. xCT contains 12 transmembrane domains with numerous conserved lysine residues. The C-terminus has three conserved lysines (K422, K472, and K473) that are positioned on the cytoplasmic surface of xCT when located on the plasma membrane, suggesting they would allow for potential interaction with trafficking proteins within the cytoplasmic compartment (Figure 1). Therefore, in this study, we sought to determine how site-directed mutagenesis of these C-terminal lysine residues affected the trafficking and activity of System x_c_^−^. This work has led to the identification of a lysine residue, K473, that appears to regulate trafficking of xCT through the ER and Golgi, and ultimately, its maturation into a functional transporter.

## 2. Results

### 2.1. Effect of C-Terminal Lysine Mutations on System x_c_^−^ Activity and Molecular Weight of xCT

As previously mentioned, xCT contains three highly conserved lysine residues located toward the C-terminus. K422 is found in a cytoplasmic loop between transmembrane domains 10 and 11, and K472 and K473 are found within the C-terminal tail (Figure 1A). These lysine sites are located on the cytoplasmic tail of xCT where they are conveniently available to undergo PTM (Figure 1B,C) and potentially interact with trafficking proteins.

**Figure 2:**
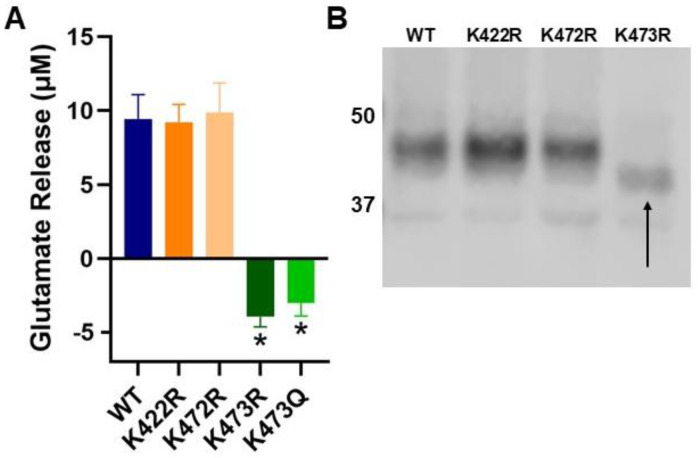
(**A**) Activity of System x_c_^−^ measured using a glutamate release assay. COS-7 cells were transiently transfected with myc-WT or mutant xCT and HA-4F2HC and glutamate release assays were performed 48 h later (*n* = 8, * *p* < 0.001 relative to WT). (**B**) Molecular weight analysis of isolated myc-WT and K → R xCT mutants transiently expressed for 48 h in COS-7 with HA-4F2HC before cell lysis. Samples were run on SDS-PAGE, blotted onto PVDF membrane and probed with mouse anti-myc antibody. WT, K422R, and K472R appear to be the same molecular weight, while K473R, as indicated with an arrow, is approximately 7.5 kD lower in molecular weight (*n* = 3).

We began this study by determining if these lysine residues (K422, K472, and/or K473) are critical for transporter activity. Since it is known that System x_c_^−^ imports cystine and exports glutamate, we measured transporter activity through a glutamate release assay. Distinct lysine to arginine (K → R) mutants were created corresponding to each of the three C-terminal lysine residues (K422R, K472R, and K473R) using site-directed mutagenesis. COS-7 cells were transiently transfected with HA-4F2HC and myc-wildtype xCT (WT) or one of the myc-K → R xCT mutants, and glutamate release assays were performed 48 h later to quantitatively measure System x_c_^−^ activity. COS-7 cells were chosen because they do not express xCT and thus have no endogenous cystine/glutamate exchange activity [1]. One-way ANOVA demonstrated that mutant type had a significant effect on transporter activity (F(5,46) = 77.38, *p* < 0.001). Specifically, K422R and K472R did not change transporter activity relative to WT, but K473R showed nearly complete loss of System x_c_^−^ activity (post hoc Tukey analysis of mutant relative to WT, *p* = 0.918, *p* = 0.995, *p* < 0.001 respectively; Figure 2A). These data suggest that K473 is critical for System x_c_^−^ function.

In order to confirm that all K → R mutants were expressed in COS-7 cells, we performed Western blot analysis of cell lysates of transiently transfected COS-7 cells. While all mutants appeared to be expressed, K473R showed a decrease in molecular weight of approximately 7.5 kD relative to WT and the other K → R mutants (Figure 2B), suggesting that K473 is either a site of constitutive post-translational modification that is necessary for proper function or K473 regulates the post-translational modification of xCT at a distinct site on the protein.

### 2.2. K473R Affects xCT Localization to the Plasma Membrane

**Figure 3:**
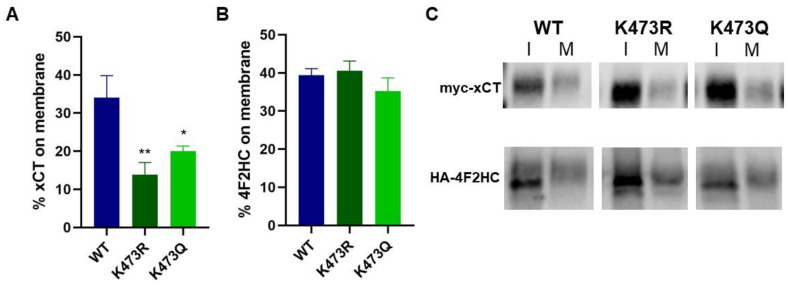
(**A**,**B**) Cell surface biotinylation assays were performed on COS-7 cells transiently expressing myc-tagged WT, K473R, or K473Q xCT and HA-4F2HC. Western blot analysis was subsequently performed to measure myc-xCT (*n* = 6) (**A**) or HA-4F2HC (*n* = 5) (**B**) abundance in intracellular and membrane fractions. Average % on membrane values are plotted (* *p* < 0.05, ** *p* < 0.001). (**C**) Representative intracellular (I) and membrane (M) bands of myc-xCT (WT or mutants) and HA-4F2HC from biotinylation assays.

Since xCT must be localized to the plasma membrane in order for System x_c_^−^ to properly function, we next sought to determine whether K473R regulated the cell surface localization of the transporter using a cell surface biotinylation assay followed by Western blot analysis. K473R showed significantly decreased membrane localization relative to WT (Figure 3A,C) (one-way ANOVA F(3,23) = 15.92, *p* < 0.001, post hoc Tukey relative to WT *p* < 0.001). These results indicate that K473 plays an important role in the trafficking and membrane localization of xCT and, therefore, the proper functioning of System x_c_^−^.

xCT must form a heterodimer with 4F2HC in order to form a functional transporter. Therefore, we also examined the effect of this mutation on the cell surface localization of 4F2HC in the same biotinylation samples. We probed the Western blots with antibodies to HA and demonstrated that nearly 40% of 4F2HC is localized to the membrane whether it is transiently co-expressed with WT xCT or K473R (Figure 3B,C) (one-way ANOVA F(3,19) = 2.861, *p* = 0.07, post hoc Tukey relative to WT *p* = 0.915). Thus, the mutation of K473 of xCT does not appear to restrict the trafficking of 4F2HC to the plasma membrane.

### 2.3. K473R Does Not Impact the Ubiquitination of the Monomer Form of xCT

Since the K473R mutation exhibited a 7.5 kD shift in molecular weight along with a significant decrease in System x_c_^−^ activity and plasma membrane localization, we sought to identify the PTM associated with K473. Lysine residues can be extensively modified in vivo. Modifications include ubiquitination (8.5 kD), neddylation (9 kD), sumoylation (12 kD), acetylation, methylation, or deamination. Ubiquitination of lysine residues regulates the trafficking of numerous membrane proteins, suggesting that ubiquitination of K473 may be necessary for the trafficking of xCT to the membrane. Moreover, given that ubiquitin has a molecular weight of 8.5 kD, which is very similar to the 7.5 kD shift in molecular weight observed for K473R, we first examined whether K473R impacted the ubiquitination of xCT.

We used anti-myc beads to immunoprecipitate WT, K422R, K472R, or K473R from transiently transfected COS-7 cells, followed by Western blot analysis using anti-myc and anti-ubiquitin antibodies. Since low doses of hydrogen peroxide (H_2_O_2_) are known to inhibit deubiquitinases, we performed this assay in transfected cells that were treated with 0.3 mM H_2_O_2_ or vehicle immediately prior to immunoprecipation in order to potentially enhance the ubiquitination of xCT.

**Figure 4:**
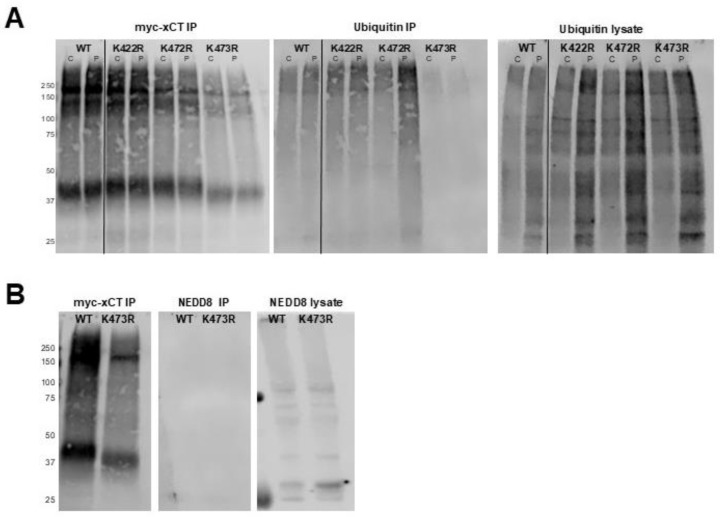
(**A**) Assessment of the ubiquitination status of xCT. Western blots of immunoprecipitated myc-xCT from transiently transfected COS-7 cells were probed with anti-myc (left) or anti-ubiquitin (middle) antibodies. COS-7 cells were treated with 0.3 mM H_2_O_2_ (P) or vehicle (C) immediately prior to collection of cell lysates and IP of myc-tagged proteins. The anti-ubiquitin signal in the original cell lysates (right) serves as a positive ubiquitin antibody control (*n* = 3). (**B**) Assessment of neddylation status of xCT. Western blots of immunoprecipitated myc-xCT from transiently transfected COS-7 cells were probed with anti-myc (left) and anti-NEDD8 (middle) antibodies. The anti-NEDD8 signal in the original cell lysates (right) serves as a positive NEDD8 antibody control (*n* = 2).

As expected, more ubiquitin immunoreactivity was observed in the lysates of H_2_O_2_-treated cells relative to vehicle-treated cells (Figure 4A). In the IP samples, ubiquitin appeared to be colocalized with xCT at high molecular weights (>100 kD), which may indicate polyubiquitination of xCT and/or xCT:4F2HC complexes. However, there was no evidence of ubiquitination of the WT xCT monomer (42 kD) or any of the K → R xCT mutants, which would be expected if xCT exhibited constitutive monoubiquitination at K473 (Figure 4A). Interestingly, K473R exhibited an almost complete loss of the high-molecular-weight ubiquitinated form of xCT, which could indicate that this mutant may not be polyubiquitinated in the same manner as WT or the other mutants. However, the data clearly demonstrated that the K473R mutation does not affect the ubiquitination status of the 42 kD monomer.

### 2.4. Neddylation Status of xCT

Neddylation is a lysine PTM, similar to ubiquitination, in which NEDD8 is covalently linked to the ε-NH_3_ group of lysine. NEDD8 has also been shown to play a role in the trafficking of membrane proteins such as the AMPA and mGlu7 receptors [33,37]. Since NEDD8 is approximately 9 kD and K473 appears to undergo a modification of approximately 7.5 kD, we sought to determine if K473 is undergoing neddylation through Western blot analysis. Similar to the ubiquitin analysis, there was no evidence of neddylation occurring on the monomer form of WT or K473R xCT, despite the fact that there was a strong NEDD8 signal in the original cell lysates (Figure 4B). Thus, the WT xCT monomer does not appear to be neddylated in vivo, and the K473R mutant does not impact the neddylation status of xCT.

### 2.5. Glycosylation Status of xCT and 4F2HC

Glycosylation occurs on over 50% of human proteins [38], playing an important role in a wide variety of cellular processes, including protein trafficking [26,39,40,41,42,43]. Glycosylation occurs on asparagine residues (N-linked glycosylation) or serine and threonine residues (O-linked glycosylation), and PTM of lysine residues has been shown to regulate the glycosylation status of several membrane proteins [34,44,45]. Moreover, the molecular weight of N- and O-glycosyl groups is variable, depending on the number of sugars attached, with glycosyl groups often ranging between 3 and 4 kD [46]. Given that xCT is predicted to contain one putative N-glycosylation site (N314) and multiple O-glycosylation sites, we reasoned that K473R may regulate the glycosylation of this transporter. Therefore, we sought to determine whether the monomer form of xCT was glycosylated.

**Figure 5:**
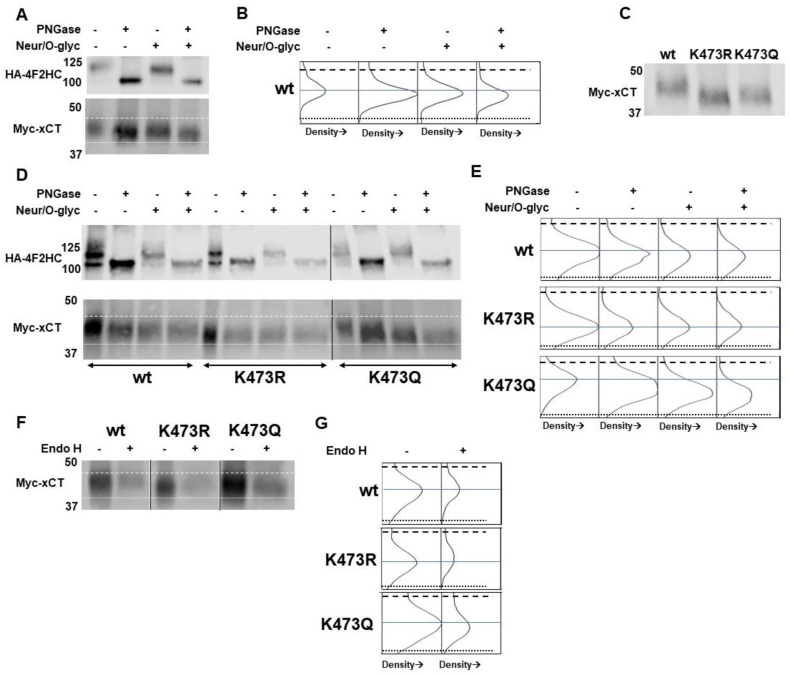
Glycosylation status of xCT and 4F2HC. (**A**) Immunoprecipitated WT myc-xCT and co-immunoprecipitated HA-4F2HC from transiently transfected COS-7 cells were treated with PNGase F, Neuraminidase/O-glycosidase (Neur/O-glyc), or all glycosidases. The monomer of xCT (bottom myc-xCT signal) shows molecular weight reductions of 2.5 kD, 1.5 kD, and 4 kD when treated with PNGase F, Neur/O-glyc, and all glycosidases, respectively. Co-immunoprecipitated 4F2HC (top HA-4F2HC signal) shows an approximate 25 kD reduction in molecular weight when treated with PNGaes F, but not Neur/O-glyc. Dashed line indicates position of the top of WT xCT band. The dotted line denotes the position of the bottom of the WT xCT band treated with all glycosidases. (*n* = 3) (**B**) Density plots for the bands of the xCT monomer when treated with PNGase F, Neur/O-glyc, and both. Pixel density increases from left to right, using the same scale for each band. Dashed and dotted lines from blot in A are added for reference. The solid blue line indicates the middle of the untreated WT band to aid in assessment of molecular weight shifts. (**C**) Molecular weight analysis of immunoprecipitated WT, K473R, and K473Q. (**D**) Immunoprecipitated WT, K473R, and K473Q and co-immunoprecipitated HA-4F2HC treated with PNGase F, Neur/O-glyc, or all glycosidases. K473R shows no molecular weight changes with glycosidase treatment. K473Q shows molecular weight reductions when treated with PNGase F, Neur/O-glyc, and all glycosidases. 4F2HC shows an approximate 25 kD loss in molecular weight upon PNGase F treatment, regardless of whether it is co-expressed with WT, K473R or K473Q xCT (*n* = 2). Dashed line indicates position of top of the WT xCT band. The dotted line indicates the bottom of the K473R band. (**E**) Density plots for WT, K473R, and K473Q. Pixel density increases from left to right, using the same scale for each band. Dashed and dotted lines from blot in D are added for reference. The solid blue line indicates the middle of the untreated xCT band for WT, K473R or K473Q to aid in the assessment of molecular weight shifts. (**F**) myc-WT, K473R or K473Q xCT immunoprecipitated from transiently transfected COS-7 cells was treated with Endo H. Only K473Q was sensitive to Endo H. (**G**) Density plots for WT, K473R and K473Q. Pixel density increases from left to right, using the same scale for each band. Dashed and dotted lines from blot in E are added for reference. The solid blue line indicates the middle of the untreated xCT band for WT, K473R or K473Q to aid in assessment of molecular weight shifts.

We treated immunoprecipitated WT myc-xCT with either Peptide-N-Glycosidase F (PNGase F), an enzyme that cleaves N-linked oligosaccharides from glycoproteins, or Neuraminidase/O-Glycosidase (Neu/O-Gly), which cleaves mucin-type O-linked oligosaccharides from glycoproteins (Figure 5A,B). We observed that PNGaseF treatment led to a 2.5 kD reduction in molecular weight of WT xCT, while Neu/O-Gly led to a modest 1.5 kD shift in molecular weight. As expected, these effects were additive, with the WT xCT sample treated with both enzymes exhibiting a 4 kD reduction relative to untreated WT, demonstrating that the xCT monomer is glycosylated in vivo.

Therefore, we next sought to determine whether K473R was also glycosylated. Given the 7.5 kD reduction in MW of K473R relative to WT, we hypothesized that K473R may not be able to be glycosylated in vivo. We treated K473R with PNGasF, Neu/O-Gly, and a combination of all glycosidases and observed no change in its molecular weight (Figure 5D,E), confirming that K473R is not glycosylated. In addition, we observed that WT xCT that was treated with both N- and O-glycosidases was still higher in molecular weight than K473R, suggesting that WT exhibits additional PTMs beyond N- and O-glycosylation under basal conditions.

We also evaluated the glycosylation status of the HA-4F2HC, which co-immunoprecipitated with myc- WT xCT and K473R. 4F2HC is a heavily glycosylated 98 kD protein, with four extracellular glycosylation sites [24,36,47]. Like many membrane proteins, the glycosylation status of 4F2HC regulates its cellular trafficking and stability [48]. Given that 4F2HC plasma membrane localization does not appear to be affected by co-expression with K473R xCT, we hypothesized that 4F2HC would continue to be glycosylated when transiently expressed with K473R in COS-7 cells. We observed that the glycosylated form (approximately 125 kD) of 4F2HC was co-immunoprecipitated with WT-xCT and K473R. Moreover, 4F2HC was sensitive to PNGase F exposure but not Neur/O–glycosidase exposure as expected. Collectively, these data demonstrate the K473R still associates with 4F2HC in vivo and that changes in the glycosylation status of 4F2HC are not responsible for the change in localization of the K473R xCT mutant.

### 2.6. Activity and Glycosylation Status of K473Q, an Acetylation Mimic Mutant of xCT

Given that K473R is unable to be glycosylated, we reasoned that K473 may regulate the glycosylation of xCT. Transient acetylation of lysine residues often occurs in the endoplasmic reticulum (ER) and plays an important role in the export of membrane proteins to the Golgi as well as maintaining protein stability [34,44]. We reasoned that if xCT could not be acetylated at K473, then perhaps it would not progress properly through the ER, precluding its ability to become glycosylated. However, acetylation at K473 may permit xCT to progress through the ER, become glycosylated and exported to the Golgi. To test this hypothesis, we created a lysine to glutamine mutant at position 473 on xCT (K473Q) and sought to evaluate whether this mutation would rescue System x_c_^−^ activity, cell surface localization and the glycosylation status of xCT. Glutamine serves as a structural mimic of acetylated lysine; thus, we predicted that this mutant would be glycosylated and exhibit comparable activity to WT when expressed in COS-7 cells. Surprisingly, K473Q also exhibited significantly decreased transporter activity (Figure 2A) and cell surface expression (Figure 3) similar to K473R. However, K473Q (41 kD) appears to exhibit an intermediate molecular weight between K473R (37.5 kD) and WT (45 kD) (Figure 5C), suggesting that it may be glycosylated.

Therefore, we performed a glycosylation analysis of the K473Q mutant to determine whether the addition of the acetyl mimetic allowed for the glycosylation of xCT. PNGaseF and Neu/O-Gly were effective in removing glycosyl groups from K473Q, demonstrating that K473Q is N- and O-glycosylated. Furthermore, when the glycosidases were used together on K473Q, the molecular weight of the mutant now matched that of the K473R mutant (Figure 5D,E). These results collectively indicate that the acetylation mimic mutant K473Q allows for the glycosylation of xCT, but glycosylation of xCT alone is not sufficient for the proper membrane localization and function of xCT.

The fact that K473Q is glycosylated but still exhibits a lower molecular weight than WT xCT may also suggest that the glycosylation of K473Q has not fully matured. Proteins are initially glycosylated in the endoplasmic reticulum, but the sugar groups are modified within the Golgi apparatus [49]. PNGase F removes all N-linked glycans by cleaving the bond between the sugar and the asparagine residue; therefore, the enzyme functions independent of the maturation state of the glycan. Endo H, however, is a glycosidase that cleaves the bond between two sugars that make up the immature glycan core. Thus, if a glycosylated protein is sensitive to Endo H, it means that the glycan has not fully matured in the Golgi and retains a structure that was originally added in the endoplasmic reticulum. Therefore, we tested the sensitivity of immunoprecipitated WT, K473R and K473Q to Endo H (Figure 5F,G). WT xCT and K473R were not sensitive to EndoH treatment, exhibiting no change in molecular weight after 3 h of treatment with EndoH. K473Q, however, exhibited a change in molecular weight such that it became equal to K473R, demonstrating that the attached glycan group was not fully matured.

### 2.7. Evaluation of Putative Glycosylation Site N314

**Figure 6:**
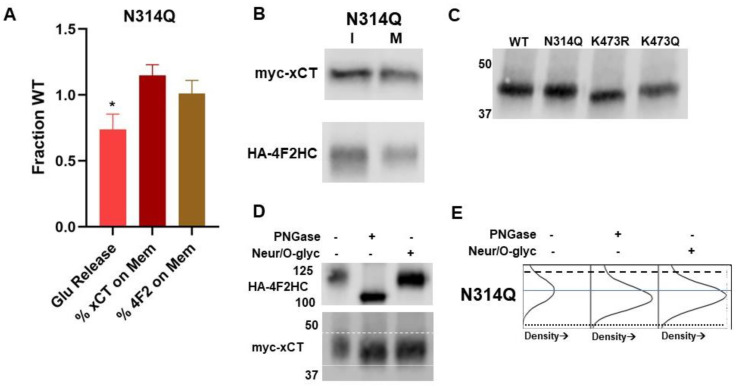
(**A**) myc-N314Q xCT was transiently expressed with HA-4F2HC in COS-7 cells and examined for function (glutamate release) and fraction localization on the membrane (biotinylation assay) relative to WT xCT. Localization of HA-4F2HC to the membrane was also measured in COS-7 cells when co-expressed with N314Q in comparison to WT. * *p* = 0.05). (**B**) Representative intracellular (I) and membrane (M) bands of myc-N314Q xCT and HA-4F2HC from biotinylation assays. (**C**) Molecular weight analysis of immunoprecipitated WT, N314Q, K473R, and K473Q. (**D**) Immunoprecipitated myc-N314Q xCT shows molecular weight reductions when treated with PNGase F and Neur/O-glyc, similar to WT. dashed line indicates the position of top of the N314Q band. The dotted line indicates the position of the bottom of the N314Q band treated with PNGaseF. Co-immunoprecipitated 4F2HC is only sensitive to PNGase F. (**E**) Density plots for the bands of myc-N314Q xCT when treated with PNGase F and Neur/O-glyc to more clearly show molecular weight shifts. Pixel density increases from left to right, using the same scale for each band. Dashed and dotted lines from blot in D are added for reference. The solid blue line indicates the middle of the untreated N314Q xCT band to aid in the assessment of molecular weight shifts.

Since we found evidence of xCT N-glycosylation, we next sought to determine whether we could identify the site of glycosylation within xCT. Asparagine 314 within xCT is predicted to be a site of N-linked glycosylation; therefore, an N → Q mutant was created (N314Q) that would remove the ability of the site to undergo glycosylation. We then assessed the effect of this mutation on transporter activity and cell surface localization. We reasoned that if glycosylation of xCT was necessary for the proper trafficking and function of this transporter, this mutation should lead to a non-functional transporter and reduced cell surface localization. However, N314Q showed much greater activity than K473R and K473Q, exhibiting 75% of the activity of WT (Figure 6A) (one-way ANOVA F(3,23) = 15.92, *p* < 0.001, post hoc Tukey *p* = 0.05 relative to WT, *p* < 0.001 relative to K473R and K473Q). Given that the activity was slightly reduced relative to WT, we next sought to determine the membrane expression of N314Q using the biotinylation assay, and it did not differ from WT (one-way ANOVA F(3,23) = 15.92, post hoc Tukey relative to WT *p* = 0.574), indicating that the partial loss of activity associated with N314Q is not due to its ability to localize to the plasma membrane (Figure 6B). Moreover, there was no effect of N314Q co-expression on 4F2HC membrane localization relative to co-expression with WT (one-way ANOVA F(3,19) = 2.861, *p* = 0.07, post hoc Tukey relative to WT *p* = 0.995). In addition, immunoprecipitation of N314Q still led to the co-immunoprecipitation of 4F2HC. Thus, the slight reduction in activity of N314Q is likely to be due to a change in the mechanics of transporter function or affinity of xCT with its substrates.

From our Western blot analysis, we observed that N314Q did not differ in molecular weight compared to WT, suggesting the N314Q is not the site of N-glycosylation as predicted (Figure 6C). To confirm this hypothesis, N314Q was transiently expressed in COS-7 cells with 4F2HC, immunoprecipitated and treated with PNGase F and O-glycosidase (Figure 6D,E). Both enzymes were effective in reducing the molecular weight of the N314Q, indicating that N314 is not a site of N-glycosylation within xCT, thus explaining why this mutation had little impact on transporter activity and membrane localization. N314Q also had no impact on the glycosylation state of the co-immunoprecipitated 4F2HC as it was still sensitive to PNGase treatment but not Neur/O-glyc treatment.

### 2.8. xCT Localization in ER and Golgi Apparatus

**Figure 7:**
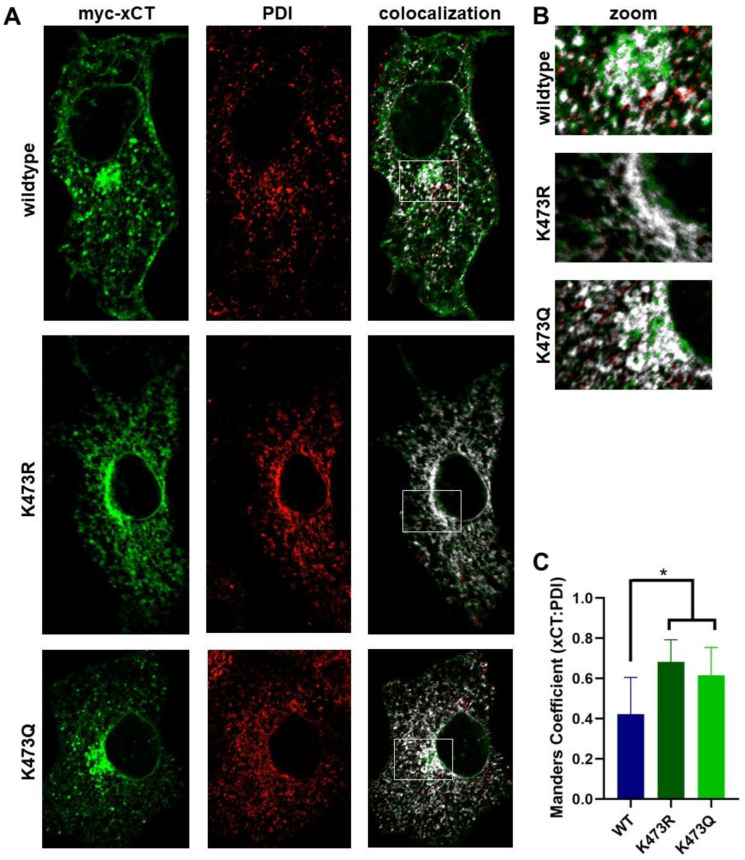
(**A**) Immunocytochemistry coupled with confocal microscopy was used to examine the colocalization of WT, K473R or K473Q xCT with PDI, an ER marker. COS-7 cells were transiently transfected with 4F2HC and WT, K473R, or K473Q for 48 h and then fixed for immunocytochemical analysis. Confocal images (single confocal section) show myc-xCT (green), PDI (red), and the Manders colocalization of xCT with PDI (white). (**B**) Zoom image of area of prominent ER density for each xCT construct indicated by white box in A. (**C**) Fraction of xCT signal that is colocalized with PDI was calculated by performing a Manders colocalization analysis (* *p* < 0.01).

**Figure 8:**
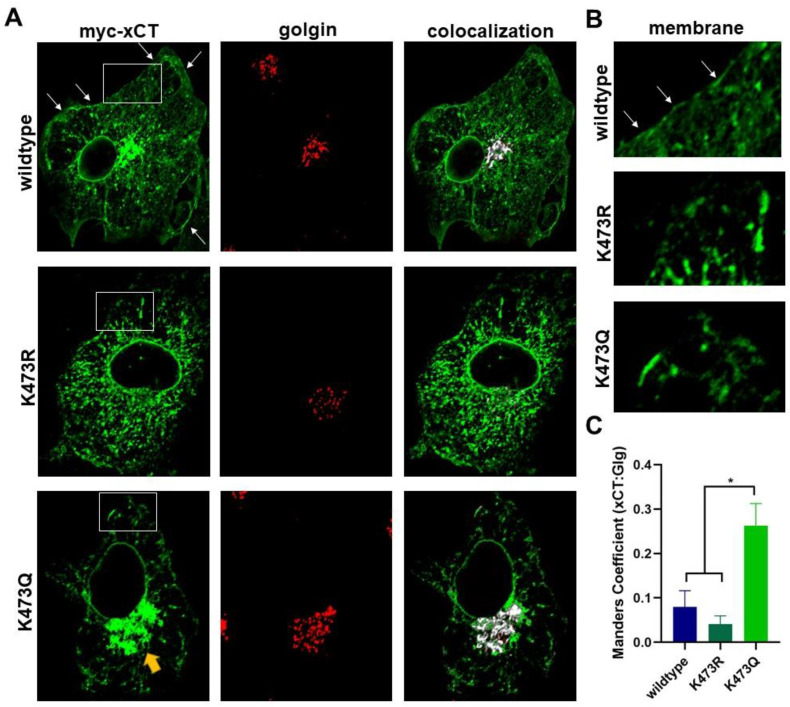
(**A**) Immunocytochemistry coupled with confocal microscopy was used to examine the localization of WT, K473R and K473Q with GM130, a Golgi marker. COS-7 cells were transiently transfected with 4F2HC and WT, K473R, or K473Q for 48 h and then fixed for immunocytochemical analysis. Confocal images (single confocal section) show myc-xCT (green), GM130 (red), and Manders colocalization of xCT with GM130 (white). Boxed areas are shown magnified in B. (**B**) Zoom image of area of membrane at the cell surface with arrows showing areas of high xCT density. These images correspond to the areas within the white box shown in A. (**C**) Fraction of xCT signal colocalized with GM130 was calculated by performing a Manders colocalization analysis (* *p* < 0.01).

Since K473R and K473Q show decreased System x_c_^−^ activity and membrane expression, we sought to use immunocytochemistry coupled with confocal microscopy to determine the intracellular localization of these mutants compared to WT. K473R is not glycosylated, a process that is initiated in the ER; therefore, we hypothesized that K473R may be retained in the ER. As expected, K473R appeared to be primarily localized around the nucleus (Figure 7 and Figure 8), with no plasma membrane expression (Figure 8). WT xCT, however, exhibited staining throughout intracellular compartments and the plasma membrane (Figure 7 and Figure 8). Using phosphodiester isomerase (PDI) as an ER marker, we observed extensive colocalization of K473R with PDI (Figure 7). We calculated the fraction of the K473R signal that colocalized with PDI by performing a Manders colocalization analysis. Compared to WT, K473R exhibited significantly higher Manders colocalization with PDI, suggesting nearly 75% of K473R was localized to the ER, while only 40% of the WT was localized in the ER (one-way ANOVA F(2,27) = 7.81, *p* = 0.002; post hoc Tukey analysis of K473R (*p* = 0.002) exhibits higher Manders colocalization with ER relative to WT).

K473Q, the acetylation mimic, is N- and O-glycosylated but exhibits reduced cell surface expression with respect to WT. In addition, it is sensitive to EndoH treatment. N-linked glycosylation matures as the protein continues from the cis-Golgi to the trans-Golgi, and most O-linked glycosylation occurs either in a translational area between the ER and Golgi or in the cis-Golgi [49]. Therefore, we hypothesized that K473Q may exit the ER but may be retained in the cis Golgi or other transport vesicles that are involved in the constitutive trafficking pathway [50]. ICC analysis of K473Q showed localization around the nucleus and a lack of plasma membrane expression, similar to K473R (Figure 7 and Figure 8). Again, significantly more K473Q co-localized with PDI relative to WT (*p* = 0.025), suggesting that the majority of the K473Q is still maintained in the ER (Figure 7).

To test our hypothesis that K473R is not exported to the Golgi but K473Q reaches the Golgi, we examined the localization of each mutant with GM130, a Golgi-specific marker. As expected, K473Q showed significantly increased colocalization with GM130 relative to WT and K473R (one-way ANOVA F(2,32) = 9.11, *p* < 0.001; post hoc Tukey test, *p* = 0.001 (WT) and *p* = 0.006 (K473R), respectively, exhibit lower colocalization than K473Q to the Golgi; Figure 8). Thus, WT xCT seems to disperse throughout the ER and plasma membrane, with very little retained within the Golgi. K473R, the acetylation deficient mutant, is withheld primarily within the ER, with virtually no colocalization to the Golgi or plasma membrane. However, K473Q, the acetylation mimic, appears to properly track through the ER and then become retained within the Golgi.

### 2.9. Effect of K473 Mutation on Association of xCT with 4F2HC

**Figure 9:**
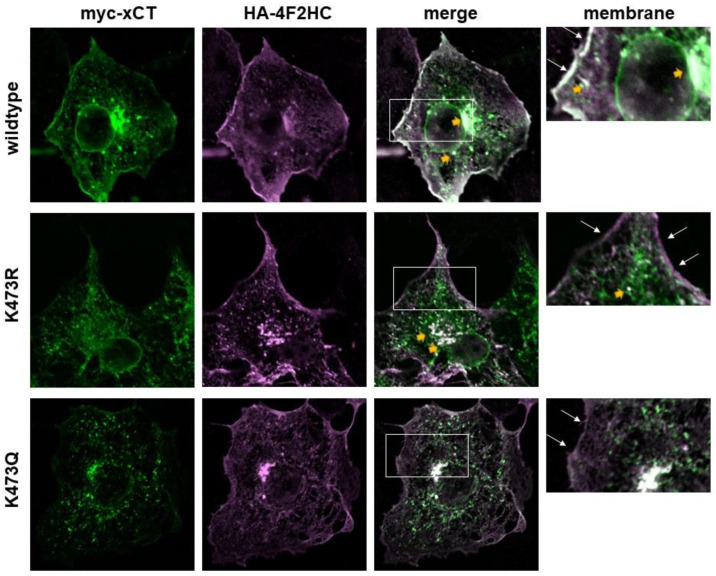
Immunocytochemistry coupled with confocal microscopy was used to examine the relative co-localization of myc-WT, K473R and K473Q xCT with 4F2HC. COS-7 cells were transiently transfected with myc-4F2HC and myc-WT, K473R, or K473Q xCT for 48 h and then fixed for immunocytochemical analysis. Confocal images (single confocal section) show myc-xCT (green), 4F2HC (purple), and merged images with areas of co-localization (white). Boxed regions in the merge images are magnified in the corresponding images in the membrane column. Thin white arrows indicate plasma membrane localization, while small wide yellow arrowsindicate regions of xCT/4F2HC co-localization within intracellular compartments.

Finally, we sought to examine the effect the K473 mutations of xCT had on its ability to traffic with 4F2HC. We previously demonstrated that K473R and K473Q xCT will co-immunoprecipitate 4F2HC and permit 4F2HC to exhibit the same degree of plasma membrane localization as co-expression with WT xCT. These are intriguing findings given that both mutants exhibit reduced plasma membrane localization, suggesting that changes in the trafficking of xCT may not impact the trafficking of 4F2HC. To address this question, we transiently co-expressed myc-WT, K473R or K473Q xCT with HA-4F2HC in COS-7 cells and examined the localization of both members of the System x_c_^−^ heterodimer using immunocytochemistry coupled with confocal microscopy (Figure 9). 

We observed that WT-xCT exhibited strong colocalization with 4F2HC along the plasma membrane, which was expected since both members of the heterodimers are required for transporter function. WT-xCT and 4F2HC also exhibited some co-localization throughout the intracellular compartments, with one notable exception that includes the area directly around the nucleus. In this region, WT-xCT can be clearly observed without an association with 4F2HC. However, as xCT extends out from the perinuclear area, it begins to exhibit greater co-localization with 4F2HC. Collectively, these observations suggest that xCT and 4F2HC associate with one another early in the biosynthetic pathway.

K473R and K473Q, however, exhibit little plasma membrane localization despite the fact that 4F2HC is highly localized to the plasma membrane, demonstrating that 4F2HC is not dependent on xCT to traffic to the membrane. Given that 4F2HC forms heterodimers with several other heteromeric amino acid transporter light chains, these results are not surprising. However, these mutants exhibit some co-localization with 4F2HC in the early biosynthetic pathway region, just distal to the perinuclear region, suggesting that they still form heterodimers with 4F2HC shortly after being synthesized. However, both K473R and K473Q exhibit distinct staining immediately around the nucleus and punctate staining throughout the cytoplasm, which does not colocalize with 4F2HC. Thus, some fractions of K473R and K473Q do not associate with 4F2HC. The lack of the ability of K473 to undergo acetylation and deacetylation in the ER and Golgi, respectively, may lead to their early degradation, suggesting that the punctate structures occupied by K473R/Q may be proteasomes or lysosomes. Future studies aimed at understanding the nature of these structures are ongoing.

## 3. Discussion

In this study, we have identified a novel mechanism for the regulation of xCT trafficking through the secretory pathway and have identified K473 as a critical residue involved in this regulatory process. Specifically, we demonstrated that mutation of lysine 473 to arginine, an amino acid of similar size and charge that cannot be post-translationally modified, eliminates System x_c_^−^ activity as a result of diminished trafficking of xCT to the plasma membrane. Instead, this transporter is sequestered nearly completely within the ER. This finding is particularly interesting because K473R appears to still be able to associate with the other member of the heterodimer, 4F2HC, which continues to traffic to the plasma membrane. Therefore, the restricted localization of K473R cannot be attributed to a dissociation from 4F2HC. Interestingly, we discovered that the K473R mutation also results in a 7.5 kD loss in the apparent molecular weight of xCT. While we originally postulated that this was a result of loss of ubiquitination or neddylation at K473, our results instead indicate that the K473R mutant lacks both N-and O-glycosylation, accounting for some, but not all, of the loss in molecular weight relative to WT. Since lysine is not a substrate for glycosylation, these results suggest that PTM of K473 may regulate the glycosylation of the transporter and its progression through the ER and ultimately prevent xCT from acquiring additional PTMs. PTM crosstalk, in which PTMs on different residues within the same protein influence each other, is a common way to meticulously regulate a protein’s activity, localization, and stability [51,52,53].

ER acetylation is a relatively recent discovery that has been found to play a crucial role in the processing of proteins in the ER that follow the secretory pathway through the Golgi and ultimately become localized to the plasma membrane [34]. In particular, ER lysine acetylation of some glycoproteins is essential for export of these proteins from the ER, proper N-glycan maturation in the Golgi, and subsequent post-Golgi trafficking [54]. For example, β-site APP cleaving enzyme 1 (BACE-1) and Prominin1 (CD133) undergo lysine acetylation in the ER lumen, a process that is necessary for their exit from the ER [44,45]. Subsequently, both proteins are deacetylated in the Golgi lumen once the protein is fully mature and trafficked to the plasma membrane. However, more recent studies have shown that at least 143 proteins are acetylated uniquely in the ER, including the System x_c_^−^ accessory protein 4F2HC [55]. While the specific mechanism by which acetylation regulates the trafficking of proteins through the ER has not been fully elucidated, recent studies suggest that neutralization of basic charges may lead to changes in ionic interactions with negatively charged phospholipids that ultimately alter the subcellular distribution of proteins [56,57].

Therefore, we tested the hypothesis that acetylation of K473 is necessary for xCT to transition appropriately through the secretory pathway by creating the structural acetylated lysine mimetic mutant K473Q. Like K473R, we discovered that K473Q lacks transporter activity and exhibits decreased cell surface localization despite still associating with 4F2HC. Unlike K473R, however, we observed that K473Q is exported to the Golgi, supporting our hypothesis that acetylation of K473 may allow for ER export, but the acetylation mimic still prevents further progression out of the Golgi. Consistent with these findings, K473Q is 3.5 kD greater in molecular weight than K473R, which we demonstrated results from its ability to be N- and O-glycosylated, similar to WT. However, unlike WT, K473Q is sensitive to EndoH glycan removal, indicating that the N-glycan is not fully mature and contains a large number of mannose sugars. Precursor N-glycans, rich in mannose, are initially added to asparagine residues in the ER, which are subsequently remodeled in the medial- and trans- Golgi, significantly decreasing the mannose content and increasing the complexity of the glycan. Thus, it is likely that K473Q is only able to proceed into the cis-Golgi compartment and is restricted from the medial- and trans-Golgi regions where maturation of N-glycans occurs. Thus, these data suggest that acetylation of K473 allows for the attachment of a precursor N-glycan to xCT in the ER, as well as export to the cis-Golgi. However, these data also suggest that deacetylation of K473 of xCT is necessary to transition out of the cis-Golgi and undergo maturation of the N-glycan, as has been noted for proteins that are acetylated in the ER [45].

Our results also suggest that WT xCT is further post-translationally modified after being exported from the Golgi. Specifically, the fully glycosylated K473Q mutant remains nearly 4 kD less in molecular weight than WT xCT and does not traffic beyond the Golgi in the secretory pathway. In addition, treatment of WT xCT with PNGase-F and O-glycosidase (41 kD) results in a deglycoslyated protein that remains greater in molecular weight than K473R and K473Q. Thus, glycosylation represents only part of the basal post-translational modifications observed on xCT. Previous research has shown that xCT can be reversibly palmitoylated, phosphorylated, and O-GlcNAcylated [28,29], suggesting that these types of PTMs may occur in the cytoplasm during post-Golgi trafficking of xCT to account for the greater molecular weight of WT xCT relative to K473Q.

**Figure 10:**
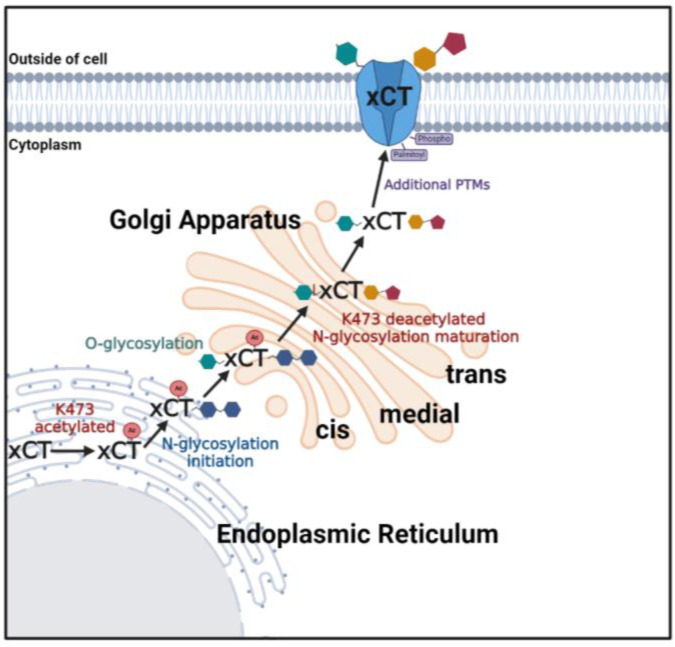
Model of K473 regulated trafficking of xCT through the biosynthetic pathway. Created in BioRender [58].

Based on these collective results, we have developed a hypothetical model that explains how K473 may regulate xCT trafficking (Figure 10) [58]. This model shares several similarities with other proteins that are known to be acetylated in the ER and deacetylated in the Golgi [1,2]. We suggest that acetylation of K473 in the ER allows for the initiation of N-glycosylation and the subsequent export of xCT from the ER into the cis-Golgi where it is O-glycosylated. Furthermore, we propose that xCT must be deacetylated at K473 in order to exit the cis-Golgi, so that the N-glycan can undergo remodeling in the medial- and trans-Golgi. Ultimately, it is exported from the Golgi within vesicles in the cytoplasmic compartment, where it can be further post-translationally modified and eventually trafficked to the plasma membrane. Given that K473R and K473Q were still able to co-immunoprecipitate 4F2HC and were shown to co-localize with 4F2HC within COS-7 cells, we do not believe that K473 regulates the interaction between xCT and 4F2HC. Therefore, we suggest that xCT and 4F2HC associate early in the ER, but, for simplicity, we have elected to omit 4F2HC from our model.

There are some important differences between the processing of xCT in the ER compared to the BACE1 and CD133; however, that must be acknowledged. (1) In the case of xCT, we find that mutation of a single lysine is able to halt progression through the ER, whereas BACE acetylates seven lysines in the N-terminus prior to ER export. (2) In addition, K473 is found within a random coil that exists within the cytoplasmic C-terminal tail. Thus, when synthesized in the ER, K473 of xCT faces the cytoplasmic compartment within the cisternae of the ER rather than the ER lumen. The acetylated lysines in BACE are localized to an extracellular region of the protein and are thus acetylated in the ER lumen. This distinction is important as it could mean that xCT is able to be acetylated by a greater array of acetylases that are localized in the cytoplasm relative to the ER. In addition, the acetylation of xCT would not be limited by ER lumenal acetyl CoA concentration as observed for BACE. (3) Finally, our data suggest that modification of K473 of xCT is necessary prior to xCT N-glycosylation while BACE is able to be glycosylated in the absence of lysine acetylation [59]. This may suggest that, unlike BACE1, K473 acetylation may be necessary for the recruitment of the oligosaccharyltransferase enzyme necessary for N-glycosylation of xCT. This finding is particularly remarkable given that the addition of the core glycan to asparagine residues typically occurs co-translationally. However, post-translational N-glycosylation has been shown to occur if the N-glycosylation site is (1) near the signal peptide sequence, (2) within 50 residues of the C-terminus, (3) close to another N-glycosylation site that is co-translationally modified, or (4) the N-glycosylation site is a part of a non-standard sequon (standard N-glycosylation sequon N-X-T/S), including those that are close to cysteine residues [60]. This final instance of post-translational modification is particularly intriguing given that the one predicted glycosylation site on xCT that exhibits the standard N-X-T/s sequon, N314, does not appear to be the site of N-glycosylation. Thus, the glycosylation site on xCT must occur on an asparagine that is not within a standard glyocosylation sequon.

Of course, we must acknowledge that more evidence is needed to develop full support for our proposed model. Most critically, we have yet to provide direct experimental evidence that K473 is acetylated in vivo. Lysine can also be modified by many small molecule metabolites including formylation, succinylation, malonylation, butyrylation, propionylation, glutarylation, β-hydroxybutylation, 2-hydroxyisobutyryation, lactylation, benzoylation and crotonylation [61]; therefore, we are currently using a mass spectroscopy approach to address this question. However, the observation that the acetylation mutant K473Q is able to transition to the cis-Golgi supports the hypothesis that K473 undergoes acetylation in the ER. In addition, the fact that the heterodimeric partner of xCT, 4F2HC, is also likely regulated by acetylation in the ER [59] would allow for effective coordination of both xCT and 4F2HC translocation through the biosynthetic pathway.

In addition, the identification of the acetylase/deacetylase enzymes that acetylate and deacetylate xCT is critically important. Given that mutation of K473 alone is able to completely halt the translocation of xCT through the secretory pathway, the identification of the acetylase that acts on K473 will allow us to understand how biosynthetic trafficking of this transporter is regulated at the cellular level. This idea is particularly intriguing given that acetylation is a key regulator of metabolism [62], especially during instances of hypoxia/reperfusion and increased generation of reactive oxygen species [63]. In other words, such a mechanism would allow for coordination of System x_c_^−^ activity and metabolic demands, allowing for increased capacity for cystine import and glutathione synthesis during times of increased vulnerability to oxidative stress. In addition, glutathione depletion increases lysine acetylation in astrocytes [64], suggesting that xCT translocation through the biosynthetic pathway could be enabled in such conditions. Such a hypothesis is consistent with our previous observation that xCT exhibits an increased rate of trafficking to the plasma membrane during glutathione depletion [1].

We also acknowledge the possibility that K473 may not be post-translationally modified in the ER and that the K473R mutation may instead disrupt protein folding to such an extent that the protein is sent immediately for degradation through the ERAD system, thus preventing it from becoming glycosylated. There is no question that K473 is highly conserved and thus must be critical for xCT function or regulation. However, given that K473 is found in a region of the C-terminus that is less ordered and relatively far from the primary transporter transmembrane cluster, which includes the cystine and glutamate translocation paths [36], it is less likely that such a minor mutation would lead to significant unfolding. Moreover, its location within a more peripheral random coiled structure is consistent with the hypothesis that this site is important for interaction with cell signaling or trafficking proteins [65]. Fractions of K473R and K473Q were localized to small puncta distributed throughout the cytoplasm, and these puncta did not exhibit colocalization with 4F2HC. Such puncta were not observed in COS-7 cells expressing WT xCT. This finding may suggest that these mutants may be targeted to either proteasomes or lysosomes for degradation once their trafficking through the biosynthetic pathway is halted. This is an area of active investigation in our lab.

Further work must also be carried out to identify the site of xCT N-glycosylation that occurs in the ER. Based on our observation that the treatment of xCT with PNGase-F led to a 2.5 kD reduction in molecular weight, there is likely only one N-glycosylation site on xCT. N-Acetylglucosamine (GlcNAc), mannose and the other monosaccharides typically found in N-linked oligosaccharides have molecular weights ranging from 0.18 to 0.22 kD/sugar. Therefore, a shift of 2.5 kD suggests between 11 and 14 sugars in the oligosaccharide, which is the typical range observed for N-linked oligosaccharides in mammals. xCT has been predicted to be glycosylated at N314 [66]; this study is the first to provide experimental evidence that xCT is N-glycosylated in vivo. However, given that the N314Q xCT mutant had the same molecular weight as WT and was sensitive to PNGaseF treatment, it is evident that N314 is not the site of N-glycosylation in this expression system. N314Q showed equivalent membrane localization as WT, despite exhibiting slightly reduced activity, suggesting that the mutation may have a modest impact on transporter function but not localization. As noted previously, we suggest that xCT is likely glycosylated at an asparagine that is part of a non-standard sequon.

Similarly, in this study, we have provided the first evidence that xCT is also O-glycosylated, a process that typically occurs in the transition between the ER and Golgi or early in the cis Golgi [49]. Neuraminidase and O-glycosidase cleave bonds between proteins and core-1- and 3-type oligosaccharides that are capped with sialic acid. They do not remove O-GlcNAc sugars that are added into the cytoplasm. Treatment of WT and K473Q xCT with these enzymes led to a 1.5 kD reduction in molecular weight, which is consistent with the expected molecular weight of core 1 O-glycosylation structures, the most common glycosylation structure types observed in humans. For example, mass spectrometry analysis of the CD8β receptor has a single core 1 O-glycosylation site to which a 1.7 kD glycan is attached, suggesting that a core 1 glycan is likely attached to xCT [67]. Thus, future work will need to probe the functional role of O-glycosylation of xCT as well as identify the site of glycosylation.

In summary, we have identified a single, highly conserved lysine residue (K473) in a cytoplasmic domain of the C-terminus of xCT that appears to regulate its progression through the ER and Golgi. Our results suggest that K473 may be transiently acetylated in the endoplasmic reticulum, allowing for the glycosylation of xCT and its export to the Golgi. Once in the Golgi, K473 likely needs to be deacetylated to allow for complete post-translational modification of xCT and its export to the plasma membrane. Thus, this site may act as a molecular switch for rapidly regulating xCT trafficking and activity.

## 4. Materials and Methods

### 4.1. Cell Culture

COS-7 cells were maintained in Dulbecco’s Modified Eagle Medium supplemented with 4 mM of L-glutamine, 4500 mg/L of glucose, 1 mM of sodium pyruvate, 1500 mg/L of NaCO_3_, 10% FBS and 1% penicillin/streptomycin in 100 mm dishes. The medium was changed every two to three days, and cultures were split 1:6 every 6–8 days. COS-7 cells were obtained from the American Type Culture Collection, and cells were not passaged more than 15 times. Tissue culture media and chemicals were purchased from Thermo Fisher (Waltham, MA, USA)

### 4.2. Human myc-xCT and HA-4F2HC Constructs

Human xCT was subcloned into pCMV-3Tag-2A (Clonetech, Mountain View, CA, USA) vector at the EcoR1/HindIII site. Human CD98 was subcloned into pCMV-HA-N (Clonetech) at the EcoR1/NotI site [1].

### 4.3. Creation of Mutants Using Site-Directed Mutagenesis

Several mutants, including K422R, K472R, K473R, K473Q, and N314Q, were created using the QuikChange Lightning Site-Directed Mutagenesis Kit (Agilent, Santa Clara, CA, USA). The QuikChange Primer Design Program website was used to design optimal primers for mutagenesis. The primer sequences are given in Table 1. All single-stranded primers were synthesized with IDT at the 25 nmol scale.

Stock primer solutions were created by resuspending each synthesized primer to a concentration of 1 mg/mL in DNase/RNase-free water. Next, working primer solutions (125 ng/µL) were prepared using the same nuclease-free water. Reagents were combined in PCR tubes according to instructions detailed in the QuikChange Lightning Site-Directed Mutagenesis Kit Manual. The template DNA used in the sample reactions was the human xCT construct (Section 4.2) purified from XL-10 Gold Competent Cells (Agilent, Santa Clara, CA, USA). The PCR reactions were run in a thermocycler (Thermo Hybaid PX2, Thermofisher, Waltham, MA, USA) following the guidelines in the QuikChange manual. Next, DpnI digestion of the template DNA and transformation of XL10-Gold ultracompetent cells were performed according to the instructions in the QuikChange manual. The transformation reaction was placed on agar plates containing kanamycin (50 µg/mL) and incubated overnight. Insertion of the correct mutations was confirmed using DNA sequencing of the entire xCT gene (Eurofins, Lancaster, PA, USA).

### 4.4. Heterologous Expression of Human myc-xCT and HA-4F2HC in COS-7 Cells

Transfection quality cDNA was produced using the Pure Yield Plasmid Midi-prep system (Promega, Madison, WI, USA). myc-xCT (wildtype or mutant) and HA-4F2HC were co-transfected into COS-7 cells using FuGENE 6 (Promega, Madison, WI, USA) following the manufacturer’s guidelines using a 3:1 ratio of FuGENE 6 to myc-xCT and a 3:1 ratio of FuGENE 6 to HA-4F2HC in an antibiotic-free medium. After 18–24 h, the medium was replaced with growth medium, which was maintained for 24 h prior to the initiation of immunoprecipitation or cell surface biotinylation assays.

### 4.5. Glutamate Oxidase and HRP Assay for Measuring xCT Activity

Activity of xCT was measured using high-throughput glutamate release as outlined by [68]. COS-7 cells grown and transiently transfected with myc-xCT (wt or mutant) and HA-4F2HC in 96-well plates were washed twice with warm PBS (37 °C) and treated with 100 µL of warm cystine (80 µM) in Earle’s Balanced Salt Solution (mM: NaCl 116.4, NaHCO_3_ 26.2, NaH_2_PO_4_ 1.02, KCl 5.36, MgSO_4_ 0.81, CaCl_2_ 1.8, glucose 5.56, pH 7.4) and incubated for one hour at 37 °C. Enzyme solution (50 µL) consisting of Glutamate Oxidase (0.04 U/mL), Horseradish Peroxidase (0.125 U/mL), Amplex (50 µM), and 1× reaction buffer (100 mM Tris, pH 7.4) was added to each well. A plate reader was used to measure the fluorescence (530 nm excitation, 590 nm emission) in each well every 30 s for 5 min in order to obtain the initial rate of glutamate turnover. A standard curve of glutamate (0–20 µM) was used to convert rates of activity to the concentration of glutamate released. Specific activity was determined by subtracting the average glutamate released (*n* = 8) from untransfected cells from the glutamate released from transfected cells.

### 4.6. Immunoprecipitation for Isolating myc-xCT

COS-7 cells transiently transfected with myc-xCT (wildtype of mutant) and HA-4F2HC were grown to 90% confluence in 35 mM dishes. Plates were placed on ice and rinsed once with ice-cold phosphate-buffered saline (PBS). Next, a cell lysis buffer (25 mM Tris, 0.15 M NaCl, 1 mM EDTA, 1% NP40, 5% glycerol; pH 7.4) containing protease inhibitor cocktail (Sigma G6521, St. Louis, MO, USA) was added to the plates. The plates were set on ice for 5 min and rocked.

The lysates were transferred to microcentrifuge tubes, centrifuged at 13,000 rpm for 10 min at room temperature, and the supernatant was transferred to new microcentrifuge tubes. Aliquots of lysate (250 μL) were added to equal volumes of Pierce Anti-c-Myc Magnetic Beads (25 μL) pre-washed with lysis buffer and incubated for 30 min at room temperature with gentle rocking.

The beads were then collected using a magnetic stand and the supernatants were transferred to new microcentrifuge tubes (unbound protein sample). The beads were washed three times with diluted 20× Mag c-Myc IP/Co-IP Buffer-2 (500 mM Tris, 3 M NaCl, 1% Tween-20 Detergent pH 7.5) and once with ultrapure water. The beads were resuspended using diluted 5× Non-reducing Sample Buffer (0.3 M Tris-HCl, pH 6.8, 5% SDS, 50% glycerol, lane marker tracking dye) and incubated for 10 min at 95 °C. The beads were collected using a magnetic stand, and the supernatants were recovered (bound protein sample). All of the samples were stored and frozen at −20 °C prior to Western blot analysis. Prior to electrophoresis, 1.3 µL of 30% SDS and 0.4 µL of β-mercaptoethanol were added to the immunoprecipitated samples to aid in xCT denaturation.

### 4.7. Biotinylation of Cell Surface Proteins

Biotinylation of cell surface proteins was performed using the method from Chase et al. with the following modification [1]. Transfected COS-7 were not serum-starved 24 h prior to the experiment, and the avidin–lysate solution was rocked for 1 h at room temperature.

### 4.8. PNGase F and EndoH to Cleave N-Linked Oligosaccharides

The immunoprecipitation protocol was followed according to Section 4.5 until resuspension of the beads. Instead of Non-Reducing Sample Buffer, the beads were resuspended in 1% RapiGest (Waters Corporation, Milford, MA, UA) in ddH_2_O. Aliquots of resuspended beads were transferred to new microcentrifuge tubes and heated at 95 °C for 10 min. For PNGase F digestion, protease inhibitors, 10× GlycoBuffer 2, and PNGase F (New England Biolabs, Ipswich, MA, USA) were added to the beads. The reaction was incubated at 37 °C for 1 h. For EndoH digestion, protease inhibitors, 10× Endo H reaction buffer, and 3 μL Endo H were added to the beads. The reaction was incubated at 37 °C for 3 h. The samples were taken for Western blot analysis and frozen at −20 °C. To prepare the samples for electrophoresis, 4× Laemmli Sample Buffer (Bio-Rad, San Francisco, CA, USA) was added to the samples, as well as β-mercaptoethanol and 30% SDS in the same ratios as in the immunoprecipitated samples in Section 4.6.

### 4.9. O-Glycosidase to Cleave O-Linked Oligosaccharides

The immunoprecipitation protocol was followed according to Section 4.5 until resuspension of the beads. Instead of Non-Reducing Sample Buffer, the beads were resuspended in 1% RapiGest (Waters) in ddH_2_O. Aliquots of resuspended beads were transferred to new microcentrifuge tubes and heated at 95 °C for 10 min. Protease inhibitors, 10× GlycoBuffer 2, α2-3,6,8 Neuraminidase, and O-Glycosidase (New England Biolabs, Ipswich, MA, USA) were added to the beads. The reaction was incubated at 37 °C for 3 h. The samples were taken for Western blot analysis and frozen at −20 °C. To prepare the samples for electrophoresis, 4× Laemmli Sample Buffer (Bio-Rad, San Francisco, CA, USA) was added to the samples, as were β-mercaptoethanol and 30% SDS in the same ratios as in the immunoprecipitated samples in Section 4.6.

### 4.10. Western Blot Analysis

Protein samples were electrophoresed at 200 V on either 12% Mini-PROTEAN TGX Precast Protein Gels (Bio-Rad, San Francisco, CA, USA) or 10% Bolt Bis-Tris Plus Mini Protein Gels (Thermo Fisher, Waltham, MA, USA) and transferred to a PVDF membrane (Millipore Immobilon-FL, Burlington, MA, USA) at 30 volts for 1 h. The membranes were blocked with Intercept (TBS) Blocking Buffer (LI-COR 927-60001, LI-COR, Lincoln, NE, USA) for 1 h at 4 °C. The membranes were probed with goat anti-myc (1:2500, Novus Biologicals NB600-335), rabbit anti-HA (1:1000, Cell Signaling #3724, Danvers, MA, USA), rabbit anti-Ub (1:1000, Cell Signaling #43124, Danvers, MA, USA) and/or rabbit anti-NEDD8 (1:1000, Cell Signaling #2754, Danvers, MA, USA) in Intercept Blocking Buffer containing 0.2% Tween 20 overnight at 4 °C. The membranes were washed in TBS-T (0.1%) 3 times for 5 min each and incubated in the dark with donkey anti-goat IRDYE 680RD (LI-COR, 926-68074, Lincoln, NE, USA) and donkey anti-rabbit IRDYE 800CW (LI-COR, 926-32213, Danvers, MA, USA), each diluted 1:15,000 in Intercept Blocking Buffer with 0.2% Tween 20 and 0.01% SDS. The membranes were washed 3 times for 5 min each in TBS-T (0.1%) and visualized using the Odyssey XF imaging system (LI-COR, Lincoln, NE, USA). Bands were also analyzed using Image Studio software v 5.2.

### 4.11. Immunocytochemistry

COS-7 cells transiently transfected with myc-xCT (wildtype or mutant) and HA-4F2HC were grown on poly-L-lysine-coated coverslips (0.01%, Sigma P4707, St. Louis, MO, USA) placed in 6 well plates. Plates were removed, placed on ice and rinsed once with ice-cold PBS, fixed with 4% paraformaldehyde in PBS for 15 min. Cells were rinsed with PBS 3 times for 2 min each. Cells were then treated with ice-cold methanol for 10 min at −20 °C and washed with PBS one more time for 5 min. In preparation for immunostaining, cells were blocked by the addition of 5% donkey sera in PBS with 0.3% Tween-20 for 1 h. Cells were incubated with primary antibody for mouse anti-myc (1:100; SCBT #SC-40, Dallas, TX, USA) or goat anti-myc (1:100; Novus, Littleton, CO, USA), rabbit anti-protein disulfide isomerase (PDI) (1:100; Cell Signaling #3501T, Danvers, MA, USA) or rabbit anti-GM130 (1:100; Cell Signaling #12480, Danvers, MA, USA), and goat anti-HA (1:100; ThermoFisher #190-138A, Waltham, MA, USA) overnight at 4 °C. Cells were washed twice with PBS and once with dH_2_O for 10 min each and then incubated with Alexa-Fluor 488 conjugated donkey anti-mouse IgG, Rhodamine RedX conjugated donkey anti-rabbit IgG (1:100), and Alexa-Fluor 647 conjugated donkey-anti goat (Jackson Immuno Research, West Grove, PA, USA) 1 h at 4 °C. The cells were washed 2 times with PBS and 1 time with H_2_O for 10 min each, and the coverslips were mounted on slides with ProLong Diamond with DAPI (ThermoFisher, Waltham, MA, USA) and dried overnight in the dark. Slides were viewed using the Nikon A1R-si confocal microscope and imaged using NIS elements imaging software version 4.60.00. Only those cells exhibiting co-expression of 4F2HC and xCT were analyzed.

### 4.12. Immunocytochemistry Co-Localization Analysis

Manders co-localization analysis was performed using the open-source software Fiji v 1.5. The background was subtracted from each color channel of an image using the rolling ball method (radius 50 pixels). The cell was outlined to create a region of interest, and the Colocalization Threshold macro was employed to determine the Manders coefficients for each cell. This macro uses the Costes et al. method [69] for determining threshold and calculating the Manders coefficients. The significance of the Manders coefficients for each cell marker and xCT was determined for mutants of xCT using one-way ANOVA followed by a post hoc Tukey test.

## Figures and Tables

**Table 1:** Forward and reverse primer sequences for the creation of xCT mutants.

Mutant	Forward	Reverse
K422R	5′-aacagtggcaccctgaaaggacgatgcatatctggg-3′	5′-cccagatatgcatcgtcctttcagggtgccactgtt-3′
K472R	5′-ttatctctttattatatgggacaggaaacccaggtggtttagaataa-3′	5′-aatagagaaataatataccctgttctttgggtccaccaaatcttatt-3′
K473R	5′-tctaaaccacctgggtctcttgtcccatataataaagagataatac-3′	5′-gtattatctctttattatatgggacaagagacccaggtggtttaga-3′
K473Q	5′-tgacattattctaaaccacctgggctgcttgtcccatataataaagagata-3′	5′-tatctctttattatatgggacaagcagcccaggtggtttagaataatgtca-3′
N314Q	5′-cggaactgctaatgagaactgtcccagtagccgctcaga-3′	5′-tctgagcggctactgggacagttctcattagcagttccg-3′

## Data Availability

The data presented in this study are available upon request from the corresponding author.
